# SWOT analysis on snail control measures applied in the national schistosomiasis control programme in the People’s Republic of China

**DOI:** 10.1186/s40249-019-0521-0

**Published:** 2019-02-06

**Authors:** Xiao Yang, Yi Zhang, Qi-Xiang Sun, Jin-Xing Zhou, Xiao-Nong Zhou

**Affiliations:** 1 School of Soil and Water Conservation, Beijing Forestry University, No.35 Qinghua East Road, Haidian District, Beijing, 100083 China; 2 Key Laboratory of State Forestry Administration on Soil and Water Conservation, No.35 Qinghua East Road, Haidian District, Beijing, 100083 China; 3 Engineering Research Center of Forestry Ecological Engineering, Ministry of Education, No.35 Qinghua East Road, Haidian District, Beijing, 100083 China; 4 National Institute of Parasitic Diseases, Chinese Center for Disease Control and Prevention, Shanghai, 200025 China; 5 Key Laboratory for Parasite and Vector Biology, National Health and Family Planning Commission, Shanghai, 200025 China; 6 WHO Collaborating Centre for Tropical Diseases, Shanghai, 200025 China; 7 Chinese Center for Tropical Diseases Research, Shanghai, 200025 China; 8 National Center for International Research on Tropical Diseases, Shanghai, 200025 China; 9 Institute of Forestry, Chinese Academy of Forestry, Beijing, 100091 China

**Keywords:** Snail control, Strategy, SWOT analysis, Chemical mollusciciding, Forestry project, Agriculture project, Water conservancy project, Schistosomiasis elimination

## Abstract

**Background:**

Snail control is an important component in the national schistosomiasis control programme in China, by application of chemical molluscicides, forestry projects, agriculture projects and water conservancy projects in recent decades. However, there are still wide areas of snail inhabited in China which remains a great challenge to achieve the goal of schistosomiasis elimination by 2025. Therefore, a SWOT (strengths, weaknesses, opportunities and threats) analysis on snail control measures is required for precision schistosomiasis control.

**Methods:**

The SWOT approach, which is a well-known structured analysis tool, was used to identify and evaluate the specific characteristics of four types of snail control measures in China, including chemical mollusciciding, forestry, agriculture, and water conservancy projects. The analysis were carried out based on the information collection from literature review, of research papers, books, annual report database of national schistosomiasis control programme in China, reports from the academic forums, and so on.

**Results:**

For chemical mollusciciding, application strategy needs to focus on specific local settings, such as stage of schistosomiasis control, environmental factors, and limitations from external policies and internal deficiencies. Regarding forestry projects, the optimal strategies are to cooperate with other national forestry programmes to share the investment costs and pay attention on wetland protection. In agriculture projects, it is necessary to develop related cash crop industries and combine with national farmland consolidation projects simultaneously to increase the total economic benefits. Concerning water conservancy projects, the main purpose is to control snail migration from snail area to snail-free areas nationwide.

**Conclusions:**

Integrated strategies for various measures application and a top-level designed cooperation mechanism will be the necessary to eliminate snail and schistosomiasis in China.

**Electronic supplementary material:**

The online version of this article (10.1186/s40249-019-0521-0) contains supplementary material, which is available to authorized users.

## Multilingual abstract

Please see Additional file 1 for translations of the abstract into the five official working languages of the United Nations.

## Background

### Schistosomiasis status in China

Schistosomiasis is an endemic disease mostly in several provinces around the Yangtze River basin and lakes in China [1]. By the end of 2017, it was estimated that there were 37 601 schistosomiasis patients in the four provinces of Anhui, Jiangxi, Hubei and Hunan. Compared with the data of more than 0.8 million cases in 2004, the number of schistosomiasis patients in 2017 had greatly decreased. Among the 450 endemic counties, 215 counties, 153 counties and 82 counties respectively reached the criteria of elimination, transmission interruption and transmission control until 2017 [2]. However, one acute schistosomiasis case reported in 2017, imported from Jiangxi to Zhejiang has indicated that the schistosomiasis epidemic situation remains transmission risks in some areas of China [3] (Table 1).

**Table 1:** The case distribution of schistosomiasis in China through 2017 [2]

Provinces	Cases of schistosomiasis japonica	Cases of acute schistosomiasis	Cases of advanced schistosomiasis
Shanghai	0	0	0
Jiangsu	2505	0	2504
Zhejiang	986	1	980
Anhui	6398	0	5631
Jiangxi	12 419	0	5000
Hubei	8434	0	8434
Hunan	4530	0	4530
Jiangxi	1	0	0
Sichuan	1689	0	1689
Yunnan	639	0	639
Total	37 601	1	29 407

### The goal of schistosomiasis elimination in China

The sixty-fifth World Health Assembly passed resolution WHA65.21, which proposed to eliminate schistosomiasis, a neglected tropical disease, in low-transmission areas of the world [4]. The conference on national schistosomiasis control in 2014 also concluded that schistosomiasis transmission will be interrupted in all endemic counties in China by 2025 [3]. Due to *Oncomelania* spp. is the unique snail intermediate host of *Schistosoma japonicum* distributed in Asia, which is an amphibious snail, so snail control is the essential factor in the process of schistosomiasis elimination, which requires a multidisciplinary cooperative mechanism and integrated implementation strategy [5, 6].

### The current distribution of *Onceomelania* spp. in China

*Oncomelania* snail distribution area increased from 351 885.06 ha to 373 596.18 ha from 2002 to 2010, respectively, representing a growth rate of 6.17% [7]. The termination of the World Bank Loan Project on Schistosomiasis Control in China (WBLP), which was in place from 1992 to 2001, resulted in a financing gap increased between the available funds and the requirements for snail control [8, 9]. Moreover, the remaining snail breeding area has continuously increased, one of reasons attributed to this increasing patterns is that the large-scale projects of pushing over embankments and returning grain fields to lakes were initiated after the extraordinary flood in 1998 [10]. In view of the risk of the spreading schistosomiasis and snails, the State Council of China implemented the Strategic Workplan for the Mid-and Long-term National Schistosomiasis Control Programme from 2004 to 2015 [11]. The main purpose of this programme is to carry out an integrated control strategy jointly via a multidisciplinary approach, involving the Ministry of Health, State Development and Reform Commission, Ministry of Finance, Ministry of Land and Resources, Ministry of Water Resources, Ministry of Agriculture and Forestry Bureau, etc., with the aim of interrupting transmission pathways and controlling the snail density and distribution [12, 13]. By the end of 2017, the total snail distribution area was 363 068.95 ha, close to that recorded in 2002 of 351 885.06 ha [1]. Therefore, there was no obvious reduction in the area of snail control from 2000 to 2017, but the snail density declined due to the implementation of multiple snail control measures, such as application of chemical molluscicides, forestry, agriculture and water conservancy projects, in the last 10 years. Nevertheless, new snail areas appeared in the recent years because snails are easily dispersed due to seasonal flooding every year [14–28]. The large snail distribution area promotes a high risk of schistosomiasis transmission in the country.

In addition, Chongqing and Henan are potential regions at risk of schistosomiasis transmission because of the Three Gorges Dam Project and the South-to-North Water Diversion Project. Large-scale hydraulic projects and human cultivation may have contributed to the snail spreading because the hydrologic conditions resulting from such projects create marshland areas where suitable for snail survival [29]. The increase of snail habitats represents a serious challenge in eliminating schistosomiasis. Determinant on improving snail control effectiveness is a critical issue in the stage of schistosomiasis elimination. Therefore, the comprehensive method of SWOT (strengths, weaknesses, opportunities and threats) analysis on various snail control measures was implemented to provide more evidences to improve integrated application strategies for the national schistosomiasis elimination programme.

## Methods

### Information collection

This study used published information from literatures to analyze the specific characteristics of four snail control measures of chemical molluscicides application, forestry, agriculture and water conservancy projects. The information for this study was based on the 88 published literatures from 1990 to 2018 in the databases including Springer Link Database (link.springer.com), China Academic Journals Full-text Database (cnki.net) and Wanfang Database (wanfangdata.com.cn), and books related to schistosomiasis prevention [30–32]. Meanwhile, this study also used data from the endemic status report of the national schistosomiasis programme of China [2, 8, 14–28], including National annual report database on schistosomiasis from 2000 to 2017 and reports of academic forums such as The Forum on Schistosomiasis control in China, Seminar collections on integrated construction technology of schistosomiasis-controlled forest. The key words for literatures search included schistosomiasis prevention, snail control measures and strategies, chemical molluscicides, snail control by forestry project, snail control by agriculture project, snail control by water conservancy project, endemic status of schistosomiasis and SWOT analysis. In addition, the research achievements from experts who are members of the National Experts Committee on Schistosomiasis Prevention were also used as key words in literature search.

### SWOT analysis

**Fig. 1:**
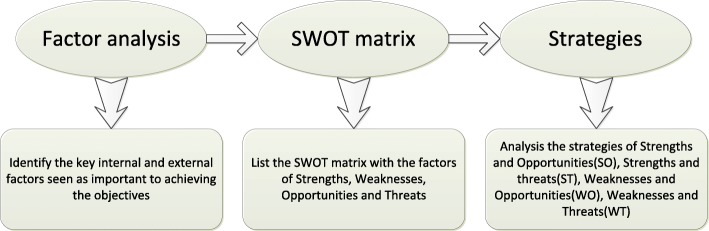
Roadmap of the SWOT analysis

The roadmap of SWOT analysis was performed by three steps, namely factor analysis, SWOT model construction and strategy development (Fig. 1). First on the factor analysis, the details of the strengths, weaknesses, opportunities and threats of chemical molluscicides application, forestry projects, agriculture projects and water conservancy projects were collected and listed. Second on SWOT model construction, we formulated matrixes of SWOT models and matched the factors with each other for system analysis to develop different strategies including a pioneering strategy with strengths and opportunities (SO), a positive strategy with strengths and threats (ST), a conservative strategy with weaknesses and opportunities (WO) and a resistive strategy of weaknesses and threats (WT). Third on strategy development, we analyzed the characteristics of the relationships between internal and external factors, with focus on how to fully make use of strengths and opportunities and avoid weaknesses and threats, in order to formulate the integrated development strategies [33].

## Results

### SWOT analysis of chemical molluscicides

Chemical molluscicides, including NaPCP, acetabromamide, niclosamide, metaldehyde, are pesticides against snail by means of releasing toxic substances [34–36]. Niclosamide is the unique molluscicides recommended by World Health Organization [37]. In reality, nicotinanilide has great advantage in rapidly killing snails, especially targeting the infested snails, and widely used in China.

#### Strengths

Chemical molluscicides such as niclosamide can be applied in various forms according to different snail distribution characteristics. For example, the wettable powder of niclosamide (WPN) is easy to use for immersion or spraying methods. The powder formulation is useful for application in water-deficient areas. Niclosamide suspensions perform better in complex environments, due to their stability. Molluscicides can kill snails in a short period, ranging from 24 h to several days. The application of 1 mg/L WPN or 0.5 mg/L for 24 h of immersion can kill all snails in one experiment [38]. The low price is a great advantage for their popularization. In general, the cost per square meter of chemical molluscicides application is RMB 0.2–0.3 [39].

#### Weaknesses

The impermanence of snail control effectiveness is the main disadvantage of the chemical mollusciciding. Due to the influence of external factors, the internally active components of molluscicides are quickly reduced, and their effect on snail control lasts only for 5–7 days [39]. Although the total area of application of chemical molluscicides was nearly 1175 thousand hectares until 2017, the existing snail distribution area still exceeded 360 000 ha in China [2]. Therefore, chemical mollusciciding do not provide permanent effectiveness in reducing areas of snail habitats. In addition, normal chemical molluscicides are toxic to other aquatic animals and produce additional environmental pollution [40]. The mechanism of action of niclosamide limits oxygen intake, influences enzymatic activity, and disrupts the physiological function of aquatic animals. Such negative results have caused economic losses and affected the development of the aquaculture industry [41]. Therefore, the development of environment-friendly molluscicides is an important research direction [42], such as a novel plant molluscicide, namely Luo-wei (tea-seed distilled saponins, TDS), has shown lethal effects on snails and less toxicity to other organisms [43–45].

#### Opportunities

The dispersed distribution of snails and complex environmental conditions have made snail control a long-term and arduous challenge in China. One document of National Action Plan to Eliminate Schistosomiasis (2016–2025) has been co-issued by several ministries of Chinese government, including National Health Commission, Ministry of Agriculture, Ministry of Water Conservation, and Ministry of Forest, etc., which will sustain the investment of schistosomiasis elimination activities [46]. In the document, it was written that the goal of schistosomiasis elimination will be achieved in all endemic area by 2025. However, by the end of 2017, among the 450 endemic counties, there have been 215, 153 and 85 counties respectively reached the criteria of elimination, transmission interruption and transmission control, indicating only 85 counties need to intensify their interventions [2]. It is essential to formulate specific strategy of molluscicides application in the stage of schistosomiasis elimination [47]. Using chemical mollusciciding is one of main strategies for reaching this target of schistosomiasis elimination, for example, schistosomiasis transmission can easily occur in newly detected snail areas or areas with infected snails where chemical molluscicides application is the optimal choice.

#### Threats

In China, the main limitation of molluscicides use is from strict laws and regulations of environmental protection. In 2015, the State Council of the People’s Republic of China issued the Water Pollution Control Action Plan aiming to control agricultural non-point-source pollution and promote the low-toxicity chemicals [48], which will limit the chemical mollusciciding in many endemic areas.

#### SWOT model

**Fig. 2:**
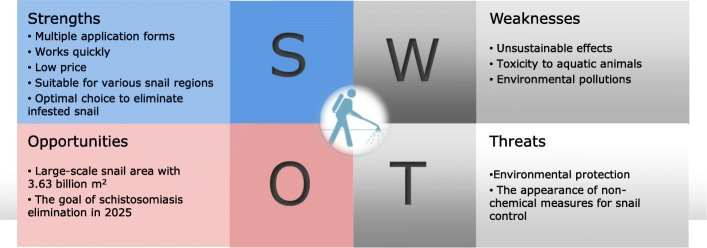
SWOT model of chemical molluscicides

Based on systematic research on the characteristics and background of chemical molluscicides application, the relevant factors were classified into the internal factors of strengths and weaknesses and, the external factors of opportunities and threats representing influential environmental factors. This classification identified positive factors representing advantages and negative factors representing disadvantages (Fig. 2).

#### Strategies

A total of four strategies were listed and described in Table 2, including pioneering strategy, positive strategy, conservative strategy and resistive strategy.

**Table 2:** Results of strategies for using chemical molluscicides

SO	1. Maintain the advantages and normal application in snail infested areas. 2. Adopt different strategies in different stages of the national schistosomiasis control programme. 3. Examine dosage forms fitted for different conditions and terrains.	Pioneering strategy
ST	1. Develop environment-friendly and targeted molluscicides to maintain the advantages.	Positive strategy
WO	1. Extend the effects of molluscicides and research slow-release options.	Conservative strategy
WT	1. Selectively apply and forbid use in environmentally sensitive areas and residential areas. 2. Coordinate with other snail control measures to achieve integrated snail control.	Resistive strategy

*SO* strengths and opportunities, *ST* strengths and threats, *WO* weaknesses and opportunities, *WT* weaknesses and threats

### SWOT analysis of forestry projects

In China, forestry projects aimed at snail control have been developed into a systematic project referred to as the Forestry Schistosomiasis Control Program. Forestry projects have been a durable and effective method for controlling snails based on altering the ecological conditions which is beneficial to snail survival [49]. According to previous studies, replacement of the original reed or weed community with plantations can alter environmental factors, such as light penetration, temperature, soil moisture and soil microorganism, and make conditions unsuitable for snail breeding [50]. In addition, forestation has changed the original structure of vegetation communities, which exhibit a favorable setting for snail breeding [51]. Moreover, research has indicated that snails can be controlled by allelopathy, substances of trees to kill snails [52].

The selection of tree species is the first step in forestry projects. The tree species should be adapted to local environmental conditions with tolerance to water-logging. Based on the allelopathic effect between trees and snails, more than one thousand species have been used in activity screening tests for snail control [53]. Several effective plant species have been identified in recent years, such as *Pterocaryastenoptera, Sapiumsebiferum, Cinnamomumbodinieri, Leonurusartemisia,* and others (Table 3).

**Table 3:** Plant species used for schistosomiasis-controlled forestry project [54]

Characteristics of snail distribution	Vegetation types	Vegetation species
Bottomland	Trees	*Populus, Salix, Taxodium ascendens, Metasequoia glyptostroboides, Taxodium distichum, Alnus cremastogyne, Pterocarya stenoptera, Sapium sebiferum, Camptotheca acuminata, Melia azedarach, Bischofia polycarpa, Lindera angustifolia, Liquidambar formosana, Taxodium hybrid ‘zhongshanshan,’ Glyptostrobus pensilis (Staunt.) Koch*
	Forbs	*Aconitum carmichaelii, Polygonum hydropiper, Leonurus artemisia, Rumex japonicus, Equisetum arvense, Calystegia hederacea, Kochia scoparia, Amaranthus retroflexus, Portulaca oleracea, Euphorbia helioscopia, Astragalus sinicus, Plantago depressa, Acorus calamus, Polygonum lapathifolium, Veratrum nigrum*
Plains with water-network	Trees	*Populus, Salix, Taxodium ascendens, Metasequoia glyptostroboides, Taxodium distichum, Melia azedarach, Toxicodendron vernicifluum, Sapindus mukorossi, Gleditsia sinensis, Pterocarya stenoptera, Sapium sebiferum, Catalpa bungei, Koelreuteria paniculata, Schima superba, Camellia oleifera, Diospyros kaki, Morus alba, Cinnamomum camphora, Ligustrum lucidum, Cupressus funebris, Toona sinensis*
	Shrubs	*Nerium indicum, Adina pilulifera, Gardenia jasminoides, Strychnos nux-vomica, Buddleja lindleyana, Magnolia liliiflora, Berberis thunbergii*
	Forbs	*Lycoris radiata, Cannabis sativa, Belamcanda chinensis, Arisaema heterophyllum, Pulsatilla chinensis, Ranunculus chinensis, Euphorbia pekinensis, Euphorbia helioscopia, Reynoutria japonica*
Mountains	Trees	*Pinus, Cunninghamia lanceolata, Quercus, Cupressus funebris, Cinnamomum camphora, Ginkgo biloba, Schima superba, Camellia oleifera, Pterocarya stenoptera, Sapium sebiferum, Platycarya strobilacea, Rhus chinensis, Toxicodendron vernicifluum, Magnolia officinalis, Pistacia chinensis, Sapindus mukorossi, Liquidambar formosana, Koelreuteria paniculata, Paulownia sieb, Cinnamomum japonicum, Ligustrum lucidum, Koelreuteria paniculata, Eucalyptus robusta, Melia azedarach, Toona ciliata, Ailanthus altissima, Ephedra equisetina, Eriobotrya japonica, Juglans regia, Castanea mollissima, Citrus reticulata, Magnolia officinalis, Eucommia ulmoides, Phellodendron amurense, Vernicia fordii, Idesia polycarpa, Zenia insignis, Toona sinensis*

#### Strengths

Above all, forestry projects aimed at snail control involve a process of ecological reform to change the original habitat. Almost no snails are found in 5-year-old and 8-year-old poplar plantations (Table 4). The forests then maintain long-term efficient snail control throughout their life span. Moreover, forestry projects can produce other benefits simultaneously, such as providing a variety of ecological services, including carbon fixation, water and soil conservation, and biodiversity protection, among others. Compared with other measures, only forestry projects can produce economic benefits for local people. Wood products and the harvest of cash crops under the forest effectively increase local annual average income [54]. Besides, forestry projects have various methods on snail control, such as allelopathy to kill snails, ecological snail control by environmental modification and altering production and life styles of local people [52, 55]. In addition, forestry project is environment-friendly snail control measure.

**Table 4:** Results of snail survey of different vegetation communities in Junshan District, Hunan province

Sampling sites	Investigation frames	Number of frames with snails	Occurring rate of snails frame(%)	Number of snails	Density of snails (number/0.11m^2^)
Sedge silvergrass community	330	310	0.94	428	1.29
Weed community	330	330	1.00	128	0.39
3-year-old poplar plantation	330	250	0.76	56	0.17
5-year-old poplar plantation	330	0	0.00	0	0.00
8-year-old poplar plantation	330	0	0.00	0	0.00

#### Weaknesses

First, forests require 3–5 years to exert sustained and steady effects on snail control. The ecological environment is basically stable in preventing snail population survival after 3–5 years [49]. Next, forestry projects have high construction costs. According to the most recent Forestry Schistosomiasis Control Program (2016–2020), the investment in forests for snail control is RMB 18000 per hectare [56]. Additionally, forestry projects have a relatively reduced scope of application compared with the use of chemical molluscicides because of their strict operation and technical regulations [57]. In addition, there are few plant species that are widely used for snail control which include poplar, willow, mulberry, and pecan. Seasonal flooding commonly provides a highly suitable area for snail survival but is not appropriate for the growth of many trees [58].

#### Opportunities

First, forestry projects are an innovative measure to reform the local environment and limit snail survival based on ecological control theory [59]. At the International Symposium on Schistosomiasis in 1992, Doctor Mott of the WHO reported that forest construction for the control of snails and schistosomiasis in China had provided a new strategy for schistosomiasis prevention to the rest of world [60]. Then, forestry projects for snail control are integrated into a multifunction program [61]. Furthermore, snail survival has seriously limited local social and economic development. In many regions near the middle and lower reaches of the Yangtze River and lakes, it is forbidden to engage in development activities because of snails and the risk of schistosomiasis transmission. Furthermore, according to the 13th Forestry Five-Year Plan, the Chinese government will initiate large-scale development of national forestry industries with the purpose of maintaining national ecological security and improving the quality of human habitats [62]. This project is a great opportunity to incorporate forestry projects for snail control into national forestry industries, with the dual benefits of forestry development and schistosomiasis prevention. There are still a large number of forests with very low-efficiency or without any effective control snail areas [63].

#### Threats

The forestry projects have also been limited by external factors. Land ownership and land use rights for forestation have influenced the development of snail control measures. The separation and interruption of ownership, management rights and land use rights have negatively influenced the forestry projects development. Moreover, certain forestation projects have the conflict with policies about wetland conservation.

#### SWOT model

**Fig. 3:**
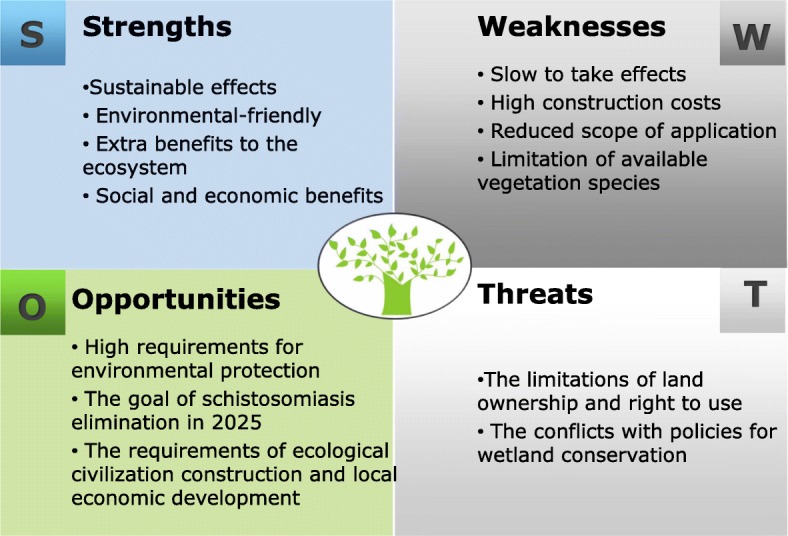
SWOT model of forestry projects

The specific content of the SWOT model for forestry projects is shown in Fig. 3.

#### Strategies

Listed below are the different strategies for developing forestry projects (Table 5).

**Table 5:** Results of the strategies for developing forestry projects

SO	1. Cooperate with the development of national forestry industries. 2. Develop the agro-forestry ecosystem. 3. Build the combination type of construction and development.	Pioneering strategy
ST	1. 1. Research new strategies for the different policies of land use and management. 2. 2. Avoid launch affforestation in the wetland protection area.	Positive strategy
WO	1. 1. Combine with molluscicides to achieve long-term and short-term solutions. 2. 2. Combine with other industries to reduce cost. 3. 3. Selectively breed vegetation with snail control effect.	Conservative strategy
WT	1. Selectively develop forestry projects in an appropriate area to avoid limitations of ecological protection policies. 2. Research new forestry project technology to improve snail control effect.	Resistive strategy

*SO* strengths and opportunities, *ST* strengths and threats, *WO* weaknesses and opportunities, *WT* weaknesses and threats

### SWOT analysis of agriculture projects

According to the investigation, 98% of snail communities were living near the upper 2 cm soil layer [49]. The main purpose of agriculture projects is to bury snails to cut off their oxygen and food resources. The different snail distribution environments are associated with different adaptable agriculture projects, such as beach cultivation, rotation between paddy fields and dry land, and terracing [64].

Beach cultivation is carried out on higher-terrain beaches during low-water-level periods in autumn. In terms of technical operations, the main aspects of this method are land smoothing, deep plowing, trenching, planting early-maturing crops or vegetation and harvesting before flood periods [65]. Rotation between paddy fields and dry land is a method to replace wet crops with dry land crops every 3 years. Decreasing soil moisture is the main purpose of rotation [64]. Terracing is an effective method for snail control on the hillsides and marshlands of mountain habitats. This technique consists of a land consolidation transforming the hillside into a smooth terrace to bury snails near the topsoil and decrease soil moisture.

#### Strengths

Agriculture projects are environment-friendly snail control measures without any associated pollution. The work involved in snail control through agriculture projects can be combined with routine farming practices and does not require extra investment. It should be noted that it is important to use appropriate agricultural measures for different snail distribution areas. Also, rotation between paddy fields and dry land can protect local people to contact with infected water.

#### Weaknesses

In general, the different agriculture projects for snail control are associated with specific technical regulations with higher cost on Capital and labors. Therefore, cost has widely limited the agricultural projects for snail control. Additionally, replacing wet crops with dry land crops in mountainous areas may result in the reduction of winter submerged field, thus affecting other agricultural production.

#### Opportunities

First, in recent years, the Chinese government has invested large amounts of funds on national farmland consolidation projects. The development of cultivation for snail control must be combined with farmland consolidation projects, especially in mountainous and hilly habitats. Moreover, farmlands with snails have always been a high-risk area for schistome infection because of the frequent contact between people and infected water.

#### Threats

Certain snail areas with broken terrain in mountain and hill habitats are usually distributed along river systems, required a large labor force, and are not suitable for mechanization. In addition, replacing wet crops with dry land crops might have the conflict with basic farmland protection policy, thus objected by local people.

#### SWOT model

**Fig. 4:**
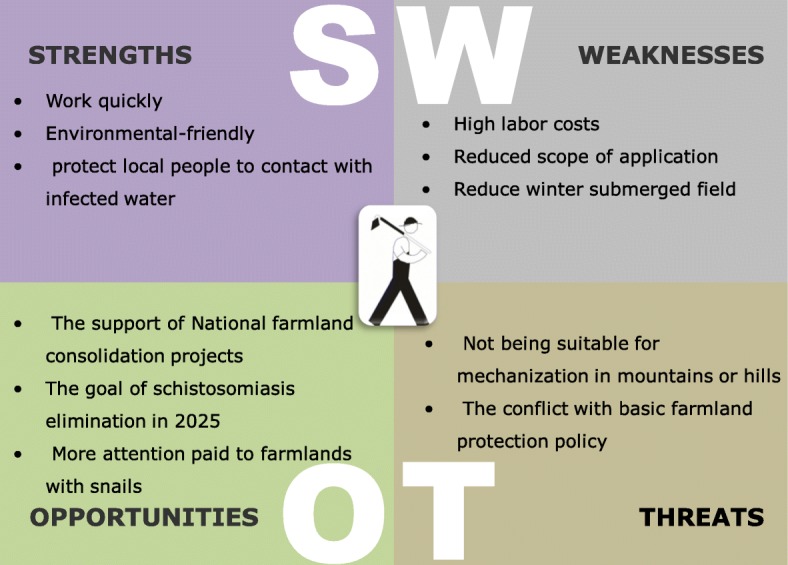
SWOT model of agriculture projects

Listed below is the specific content of the SWOT model for agriculture projects (Fig. 4).

#### Strategies

As for the above section, the different strategies for developing agriculture forestry projects are described in Table 6.

**Table 6:** Results of the strategies for developing agriculture projects

SO	1. Build a cooperative mechanism between health and agriculture administrations. 2. Develop the agro-forestry ecosystem and agricultural economy under trees to support agriculture projects for snail control.	Pioneering strategy
ST	1. Selectively develop agriculture projects in appropriate areas to avoid terrain limitations. 2.Selectively develop replacing wet crops with dry land crops to avoid limitation of basic farmland protection policy	Positive strategy
WO	1. Coordinate with the policies for agriculture development, such as agricultural farmland construction, to lower the cost. 2. Coordinate with other measures to interrupt new snails spread into the farmland.	Conservative strategy
WT	1. Combined with forestry projects to form ecological isolation zone between farmland and rivers. 2. Develop with cash crop planting to increase economic benefits.	Resistive strategy

*SO* strengths and opportunities, *ST* strengths and threats, *WO* weaknesses and opportunities, *WT* weaknesses and threats

### SWOT analysis of water conservancy projects

Water is necessary for snail survival and is the main source of snail spreading [49]. Additionally, infected water provides the opportunity for schistosomiasis to penetrate human skin while in contact with it. Therefore, water bodies such as lakes, rivers, irrigation canals and ditches have played important roles in schistosomiasis transmission [66]. There are a number of water conservancy projects for snail control, involving approaches such as hardening the banks of rivers and lakes; constructing isolated canals, culverts and sluice gates; constructing snail retention reservoirs; middle layer water intake; and lining of irrigation ditches with cement [66].

#### Strengths

First, water conservancy projects for snail control can effectively decrease the mobility of snail populations because flooding has always been the main source of snail diffusion. Then, water conservancy projects can improve the sanitary conditions of local residents by hardening the banks of rivers or lakes and lining of irrigation ditches or canals with cement [67]. The construction of water conservancy projects is convenient for farm irrigation, which increases the benefits of agricultural production [68]. These projects greatly improved the quality of the living environment near rivers or lakes and maintain a snail-free environment for local people [69]. Moreover, water conservancy projects can play an important role in controlling snails spread over a long distance. The large water conservancy projects such as the Three Gorges Project and the South-to-North Water Diversion Project are national long-term investment projects [70]. Therefore sustainable development strategy is to construct snail control facilities combined with large conservancy projects.

#### Weaknesses

First, the investment required for water conservancy projects is far beyond ability of most local governments regarding hardening the banks of rivers and lining of irrigation ditches with cements. Furthermore, there are substantial contradictions between water conservancy projects and ecological environmental protection. Hardening banks and ditches with cements has cut off the connectivity of biotic communities [71]. The concept of ecological theory involves maintaining natural states and reducing human interference. But the natural banks of rivers, lakes and natural canals are suitable for snail survival. Therefore, maintaining the balance between snail control projects and ecological management is necessary. In addition the main functions of the normal water conservancy projects will interrupt the spread of snails, but not eliminate them [72].

#### Opportunities

Certain structures related to water conservancy projects, such as irrigation ditches, are components of national farmland consolidation projects, providing great opportunities to develop facilities for snail control. Additionally, for culvert and sluice gates, snail retention reservoirs and middle-layer water intake are necessary in large water conservancy projects, in which snail possibly diffuse with water [67].

#### Threats

According to ecological theory, lining of ditches with cement and the hardening of river banks negatively influence the balance of the hydrologic cycle, biological diversity and the stability of ecosystems [73]. However, natural conditions without artificial measures provide a suitable environment for snail survival, seriously threatening the health of local people. For water conservancy projects, the conflict between snail control and ecological protection will be a great challenge in future development [72].

#### SWOT model

**Fig. 5:**
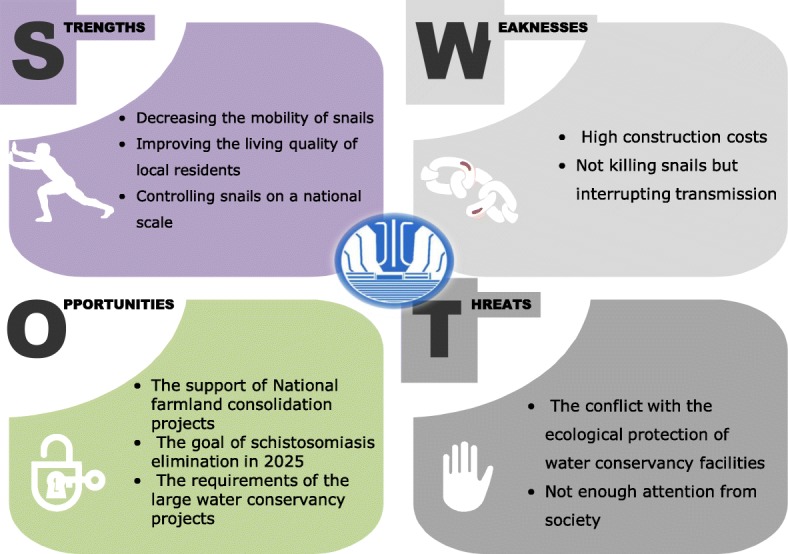
SWOT model of water conservancy projects

The specific content of the SWOT model for water conservancy projects is provided in Fig. 5.

#### Strategies

The different strategies for developing water conservation are as follows (Table 7).

**Table 7:** Results of the strategies for developing water conservancy projects

SO	1. Incorporate snail control buildings or facilities into large water conservancy projects such as the Three Gorges Project and the South-North Water Diversion Project to prevent the remote migration of snails. 2. Promote the use of cement lining of irrigation ditches in national farmland consolidation projects.	Pioneering strategy
ST	1. Develop educational activities to disseminate knowledge about snail control through different types of water conservancy projects and raise awareness among local people. 2. Develop novel measures for water conservancy projects to coordinate with theories of ecological protection.	Positive strategy
WO	1. Cooperate with the national farmland consolidation projects and other large water conservancy projects to lower cost. 2. Combine with other measures for snail control and form an integrated snail control system.	Conservative strategy
WT	1. Selectively develop water conservancy projects in appropriate areas and avoid destroying the ecological preservation areas. 2. Research new technology to reduce the required investment.	Resistive strategy

*SO* strengths and opportunities, *ST* strengths and threats, *WO* weaknesses and opportunities, *WT* weaknesses and threats

## Discussion

### Pioneering strategy

Chemical molluscicides are main measure in the newly detected snail area, which can eliminate snails, especially infested snail, quickly to decrease the risk of schistosomiasis retransmission again in the regions with the criteria of transmission interruption and elimination [27]. Meanwhile, it is necessary to develop various dosage forms, such as powder, granules, suspension concentrate and retarder, adopting different local situation of snail areas [74, 75].

Developing agro-forestry ecosystem always is the important strategy to maintain the balance between snail control and economic benefits [60]. Sustained economic benefit is the vital force to promote the sustainable development of Forestry Schistosomiasis-control Program. Meanwhile, the ecological isolation strips integrated technical means from agriculture and forest projects also are effective measures to stop snail spreading. In addition, projects of agriculture, forestry and water conservancy need to be combined with related national constructions, such as Agricultural Farmland Construction, national greening campaign and large-scale water conservancy projects, to get more space for development.

Furthermore, it is necessary to carry out continuous quality and benefits monitoring for the implementation of different control measures. Beginning from 2018, The World Bank Loan Project Management Center of State Forestry and Grassland Administration have developed the quality and benefits monitoring project of Forestry Schistosomiasis Control Programs in the next 3 years. The project has built several long-term monitoring points in Hubei, Anhui and Jiangxi provinces. Specific contents included endemic status, snail distribution pattern, snail density, environmental factors, forest areas, forest stand quality, construction and management. The monitoring and analysis on these key indexes were able to objectively evaluate the quality of Forestry Schistosomiasis Control Programs and further provide data support for optimization and upgrading. Therefore, administrations of molluscicides, agriculture and water conservancy projects also should develop the relative monitoring plan for accumulating data and create more possibilities on technology promotion and cooperation with others.

### Positive strategy

Different snail control measures also need adapt the relative national policies. Chemical molluscicides application must be accord with environmental protection. And then, environment-friendly molluscicides should be researched to expand application scope. Similarly, forestry, agriculture and water conservancy projects are necessary to adapt the policies of wetland protection, environmental protection of Yangtze River economic belt and ecological restoration [72, 76].

Besides, more sensitive techniques of snail surveillance should be developed to improve the precision of snail survey and snail control efficiency in epidemic and potential areas [77, 78]. A series of studies have indicated that the effective accumulated temperature was able to satisfy snail and schistosome to complete a growth cycle in some regions in the north of China [79]. Therefore, global warming may cause a risk of snail spreading northward. The intake of South-to-North Water Diversion Project is located in the epidemic area of schistosomiasis [80, 81]. In addition, the construction of Three Gorges Project and the South-to-North Water Diversion Project has newly formed large-scale areas fitted for snail survival [29]. Thus a dynamic monitoring and warning systems, being able to predict and evaluate the variation of environmental factors in snail distribution areas, need be built to improve the sensitivity of snail detection and offer the data to support the goal of schistosomiasis elimination [82].

In addition, researching the relation model in regard to snail density, snail areas and schistosomiasis transmission is able to quantitatively analyze the threshold value of snail distribution and provide foundation for decision-making. Former statistics data showed that the cases of schistosomiasis have decreased from 756 762 to 37 601 and acute schistosomiasis also decreased significantly from the year of 2000 to 2017. But the decline of snail areas is not obvious. The change of snail area has possibly no longer reflected the effect of schistosomiasis prevention [1].

Moreover, the systematic technology export on snail control measures has been the great opportunities to develop and promote Chinese experiences for the assistance to schistosomiasis endemic area in Africa and Southeast Asia [83]. In 2014, China, Zanzibar and WHO have officially signed the memorandum of understanding on the cooperative pilot project of schistosomiasis prevention [84]. At present, applying molluscicides still is the main measure on snail control in Africa. Other techniques including forestry, agriculture and water conservancy projects have a great opportunity to develop in Africa. The output of snail control techniques also provided the possibility to combine with local situation in African creating a new space aim at self-optimization and improvement of snail control measures [85].

### Conservative strategy

Regarding to chemical molluscicides, the characteristics of long-term and slow-release is the key technical problems in future research filed. Years of statistics data indicated that repeated chemical molluscicides application has failed to decrease snail areas in China because of the short-term effect [2, 8]. Furthermore, breeding the appropriate species with molluscicidal compounds and water resistant also are the important research filed for forestry project. For agriculture and water conservation project, the further strategies are to decrease the construction costs by researching new technology and materials [86].

### Resistive strategy

In the face of various limitations during the actual application process, selective application also is a kind of strategy. Chemical molluscicides should be forbid to apply in the areas of environmental sensitive and residential living [42]. Also, forestry projects have to be constructed outside the wetland reserves aimed at wetlands protection [87, 88]. For the forest of low schistosomiasis-controlled function, it is necessary to carry out transforming and upgrading operation based on the technical regulations for improvement of snail control effect through the schistosomiasis prevented forestry project [56]. Besides, the administrations of agriculture and poverty alleviation should build a coordination mechanism to advocate captive livestock aimed at avoiding schistosomiasis transmission by means of cattle or other livestock. For water conservancy project, it is necessary to keep the balance of ecological restoration and snail control [86]. The optimal strategy is to improve quality and efficiency of technologies.

The limitation of this study is that there are no formatted feasible technological regulations for actual execution, but only strategic research. Thus, it will be necessary to begin more surveys and analysis work in the future and to progressively develop a complete theoretical system and technical guide for integrated snail control.

## Conclusions

This study has analyzed four snail control measures from strengths, weaknesses, opportunities and threats, as well as put forward multiple application strategies. Chemical molluscicides application is a convenient approach to eliminate infested snails quickly without geographical limitations. In addition, Forestry project is a kind of long-term and environment-friendly snail control measure with additional economic benefits. Furthermore, agriculture project can completely alter original production and life style and protect local people contacting with infested water. Moreover, the characteristics of water conservancy project are to interrupt snail migration and contribute to agricultural production by mean of hardening ditches and channels. Every measure is irreplaceable on snail control in China. Also, it is necessary to build the top-level design for the cooperation mechanism and integrated snail control measures including the resources of sanitation, forestry, agriculture and water conservancy [29, 89].

## Additional file

Additional file 1: Multilingual abstracts in the five official working languages of the United Nations. (PDF 577 kb)

## Abbreviations

NaPCP: Niclosamide, sodium pentachlorophenate
SO: Strengths and opportunities
ST: Strengths and threats
SWOT: The strengths, weaknesses, opportunities and threats
WHA: World Health Assembly
WHO: World Health Organization
WO: Weaknesses and opportunities; WT: weaknesses and threats
WPN: Wettable powder of niclosamide
