# Supplementary material for: SWOT analysis on snail control measures applied in the national schistosomiasis control programme in the People’s Republic of China
Source: Infect Dis Poverty. 2019 Feb 6;8:13. doi: 10.1186/s40249-019-0521-0 (PMC6367817; doi:10.1186/s40249-019-0521-0)
Supplement: Supplementary file 1 — Multilingual abstracts in the five official working languages of the United Nations. (PDF 577 kb) [file 40249_2019_521_MOESM1_ESM.pdf]

Translation of the abstract into the five official working languages of the United Nations

تحليل SWOT (نقاط القوة والضعف والفرص والأخطار) لإجراءات مكافحة القواقع المطبقة في البرنامج الوطني لمكافحة البلهارسيا بجمهورية الصين الشعبية

شياو يانج، يي تشانج، كي شيانج صن، جين شينج تشو، شياو نونج تشو

ملخّص
خلفية: تعد مكافعة القواقع عنصرًا مهمًا في البرنامج الوطني لمكافحة البلهارسيا بالصين عن طريق استخدام مبيدات رخوية كيميائية، ومشاريع الغابات، والمشاريع الزراعية، و مشاريع حفظ المياه في العقود الأخيرة. ولكن، لايزال هناك العديد من المناطق التي تسكنها القواقع بالصين، والتي تبقى تحديًا يواجه تحقيق هدف القضاء على البلهارسيا بحلول عام 2025. ولهذا؛ يتطلب إجراء تحليل SWOT (نقاط القوة والضعف والفرص والأخطار) على إجراءات مكافحة القواقع للسيطرة الدقيقة للبلهارسيا
المناهج: تم استخدام طريقة تحليل SWOT - التي تعد أداة تحليل منظمة مشهورة - لتحديد، وتقييم الخصائص المحددة لأربعة أنواع من إجراءات مكافحة القواقع بالصين، والتي تتضمن المبيدات الكيميائية للرخويات، والغابات، والزراعة، ومشاريع حفظ المياه. وقد أُجري التحليل بناء على جمع المعلومات من مراجعة مطبوعات الأوراق البحثية، والكتب، وقاعدة بيانات التقارير السنوية للبرنامج الوطني للقضاء على البلهارسيا في الصين، والتقارير الواردة من المحافل الأكاديمية، وما إلى ذلك.
النتائج: بالنسبة للإبادة الكيميائية للرخويات يتعين على استراتيجية التطبيق التركيز على إعدادات محلية محددة؛ مثل مرحلةمكافحة البلهارسيا، والعوامل البيئية، والحد من السياسات الخارجية وأوجه القصور الداخلية. وبالنسبة لمشاريع الغابات، فالاستراتيجيات المثلى هي التعاون مع برامج وطنية أخرى للغابات من أجل المشاركة في تكاليف الاستثمار، والاهتمام بحماية الأراضي الرطبة. وبالنسبة للمشاريع الزراعية؛ فإنه من الضروري أن يتم تطوير صناعات محاصيل نقدية متعلقة بالأمر، والتعاون مع المشاريع الوطنية لدمج الأراضي الزراعية بشكل متزامن لزيادة العوائد الاقتصادية الإجمالية. وبالنسبة لمشاريع حفظ المياه؛ الهدف الأساسي هو مكافحة هجرة القواقع من المناطق العامرة بالقواقع إلى المناطق الخالية منها في جميع أنحاء الدولة.
الاستنتاجات:الاستراتيجيات المدمجة لتطبيق الإجراءات المختلفة وآليات التعاون المصممة رفيعة المستوىستكون ضرورة للقضاء على القواقع والبلهارسيا بالصين.

Translated from English version into Arabic by Duaa Elsharkawy, proofread by Amal Imam, through

TRANSLATORS
WITHOUT BORDERS

中国血吸虫病防治工作中钉螺控制措施的 SWOT 分析

杨筱, 张仪, 孙启祥，周金星, 周晓农

摘要
引言：钉螺控制是中国血吸虫病防治工作的重要组成部分。几十年来，药物灭螺、农业血防工程、林业血防工程和水利血防工程等多种钉螺控制措施综合应用，是中国血吸虫病防治的主要策略。然而，大面积钉螺分布对于 2025 年全国消除血吸虫病仍是一项巨大的挑战。因此，精准消除血吸虫病需要对钉螺控制措施进行 SWOT 分析。
方法：本研究采用 SWOT 分析法对 4 种钉螺控制措施，即药物灭螺、农业血防工程、林业血防工程、水利血防工程的具体特征进行分析与评估。相关的数据和资料来源包括各类研究性和综述性文献、近十多年的血吸虫病年报数据库、疫情通报和血防学术会议的报告等。
结果：药物灭螺应针对不同地域特征、环境因素和血吸虫病防治阶段等特点，结合自身特性，开展精准的靶向灭螺。林业血防工程应与国家重点林业项目有机结合，分担投资成本。同时优先考虑湿地资源保护，避免造林

对湿地生态系统的影响。此外，农业血防工程应重点发展相关的经济作物产业模式，与国家土地整治项目相结合，抑螺防病和经济效益提升相辅相成。水利血防工程应侧重于大型水利工程中抑螺成效提升，防止钉螺由有螺区扩散至无螺区。
结论：实施综合防控策略并且通过顶层设计建立合作共享机制，才能真正助力于血吸虫病消除目标的顺利实现。

Translated from English version into Chinese by Xiao Yang

Analyse SWOT des mesures de contrôle des populations d'escargots mises en œuvre dans le cadre du programme national de lutte contre la schistosomiase en République populaire de Chine

Xiao Yang, Yi Zhang, Qi-Xiang Sun, Jin-Xing Zhou, Xiao-Nong Zhou

Résumé

Contexte : Le contrôle des populations d'escargots, au moyen de molluscicides chimiques et de projets forestiers, projets agricoles et de conservation des eaux, est une composante importante du programme de lutte contre la schistosomiase en Chine depuis quelques décennies. Des populations d'escargots hôtes subsistent cependant dans de vastes zones du pays et constituent un obstacle majeur pour atteindre l'objectif d'élimination de la schistosomiase d'ici à 2025. En conséquence, une analyse SWOT (strengths, weaknesses, opportunities and threats – forces, faiblesses, opportunités et menaces) des mesures de lutte contre les escargots est requise pour une lutte plus précise contre la schistosomiase.
Méthodes : La méthode SWOT, qui est un outil d'analyse structurée très répandu, a été utilisée pour identifier et évaluer les caractéristiques spécifiques de quatre types de mesures de lutte contre les escargots en Chine: épandage de molluscicides chimiques, projets forestiers, projets agricoles et projets de conservation des eaux. Notre analyse s'est basée sur des informations recueillies par l'analyse de revues de la littérature, d'articles de recherche, de livres et de la base de données des rapports annuels du programme national de lutte contre la schistosomiase en Chine, de rapports universitaires, etc.
Résultats : Pour l'utilisation de molluscicides chimiques, la stratégie d'épandage doit être adaptée au contexte local, par exemple à la phase de la lutte contre la schistosomiase, aux facteurs d'environnement et aux limites imposées par les politiques externes et les faiblesses internes. Concernant les projets forestiers, les stratégies optimales consistent dans la coopération avec d'autres programmes forestiers nationaux en vue de mutualiser les investissements et dans une meilleure protection des milieux humides. Dans les projets agricoles, les cultures de rente doivent être développées et combinées simultanément avec des projets nationaux de remembrement des terres agricoles en vue d'augmenter les bénéfices économiques totaux. Concernant les projets de conservation de l'eau, l'objectif principal est la prévention, à l'échelle nationale, de la migration des escargots des zones qu'ils occupent vers d'autres zones où ils ne sont pas encore présents.
Conclusions : Des stratégies intégrées pour la mise en œuvre de diverses mesures et un mécanisme de coopération de haut niveau seront nécessaires pour éliminer les escargots hôtes et la schistosomiase en Chine.

Translated from English version into French by Ingrid Vieira Mary, proofread by Suzanne Assenat, through

TRANSLATORS
WITHOUT BORDERS

SWOT-анализ мер борьбы с улитками, применяемых в рамках государственной программы борьбы с шистосомозом в Китайской народной республике

Сяо Янь, Йи Чжан, Ци-Сянь Сунь, Цзинь-Синь Чжоу, Сяо-Нонг Чжоу

Аннотация

Общие сведения: в последние десятилетия в Китае борьба с улитками является важным компонентом государственной программы борьбы с шистосомозом путем применения химических моллюскоцидов в лесохозяйственных, сельскохозяйственных проектах и проектах по охране водных ресурсов. Тем не менее, в Китае по-прежнему существуют обширные районы обитания улиток, что остается большой проблемой для ликвидации шистосомоза к 2025 году. Поэтому для точного контроля уровня распространения шистосомоза необходим SWOT-анализ (сильные и слабые стороны, возможности и угрозы) мер по борьбе с улитками.

Методы: подход SWOT – это хорошо известный инструмент структурированного анализа, использующийся для выявления и оценки конкретных характеристик четырех типов мер по борьбе с улитками в Китае, и включающий проекты в сфере химического моллюскицидирования, лесного хозяйства, сельского хозяйства и охраны водных ресурсов. Анализ осуществлялся на основе обзора литературы, научных трудов, книг, годовых отчетов базы данных Национальной программы по борьбе с шистосомозом в Китае и репортажей с научных форумов и т.д.

Результаты: для химического моллюскицидирования стратегия применения должна учитывать специфические местные условия, такие как стадия борьбы с шистосомозом, факторы окружающей среды, а также условия и ограничения внешней и внутренней политики. Что касается лесохозяйственных проектов, то оптимальными стратегиями являются сотрудничество с другими национальными лесохозяйственными программами в целях распределения инвестиционных расходов и охраны водно-болотных угодий. В сельскохозяйственных проектах необходимо развивать смежные отрасли товарного растениеводства и одновременно сочетать их с государственными проектами консолидации сельскохозяйственных угодий для увеличения общих экономических выгод. Что касается проектов сохранения водных ресурсов, то основной целью является контроль миграции улиток из мест их обитания в районы, свободные от улиток по всей стране.

Выводы:для ликвидации улиток и шистосомоза в Китае необходимы комплексные стратегии применения различных мер и разработанный на высшем уровне механизм сотрудничества.

Translated from English version into Russian by Michael Orlov, proofread by Tatiana Karymshakova, through

TRANSLATORS
WITHOUT BORDERS

Análisis FODA de las medidas de control de caracoles aplicadas en el programa nacional de control de la esquistosomiasis en la República Popular de China

Xiao Yang, Yi Zhang, Qi-Xiang Sun，Jin-Xing Zhou, Xiao-Nong Zhou

Resumen

Fondo: El control de caracoles es un componente importante en el programa nacional de control de la esquistosomiasis en

China, mediante la aplicación de molusquicidas químicos, proyectos forestales, proyectos agrícolas y proyectos de conservación del agua en las últimas décadas. Sin embargo, todavía hay áreas extensas de caracoles habitadas en China, que siguen siendo un gran desafío para lograr el objetivo de la eliminación de la esquistosomiasis en 2025. Por lo tanto, se requiere un análisis FODA (fortalezas, debilidades, oportunidades y amenazas) sobre las medidas de control de caracoles para lograr controlar una esquistosomiasis precisa.

Métodos: El enfoque FODA, que es una herramienta de análisis estructurado bien conocida, se utilizó para identificar y evaluar las características específicas de cuatro tipos de medidas de control de caracoles en China, incluidos los proyectos de molusquicio químico, silvicultura, agricultura y conservación del agua. El análisis se realizó sobre la base de la recopilación de información de la revisión de la literatura, los trabajos de investigación, los libros, la base de datos de informes anuales del programa nacional de control de la esquistosomiasis en China, los informes de los foros académicos, etc.

Resultados: Para los molusquicidas químicos, la estrategia de aplicación debe centrarse en entornos locales específicos, como la etapa de control de la esquistosomiasis, los factores ambientales y las limitaciones de las políticas externas y las deficiencias internas. Con respecto a los proyectos forestales, las estrategias óptimas son cooperar con otros programas forestales nacionales para compartir los costos de inversión y prestar atención a la protección de los humedales. En los proyectos agrícolas, es necesario desarrollar industrias de cultivos comerciales relacionadas y combinarlas con proyectos nacionales de consolidación de tierras agrícolas simultáneamente para aumentar los beneficios económicos totales. Con respecto a los proyectos de conservación de agua, el objetivo principal es controlar la migración de caracoles desde el área de caracoles a áreas sin caracoles en todo el país.

Conclusiones: Las estrategias integradas para la aplicación de varias medidas y un mecanismo de cooperación diseñado de alto nivel serán las necesarias para eliminar el caracol y la esquistosomiasis en China.

Translated from English version into Spanish by Lia Sarra Felip, proofread by Elena Tentoma, through

TRANSLATORS
WITHOUT BORDERS
